# *Plasmodium* Rab5b is secreted to the cytoplasmic face of the tubovesicular network in infected red blood cells together with N-acylated adenylate kinase 2

**DOI:** 10.1186/s12936-016-1377-4

**Published:** 2016-06-17

**Authors:** Kazuo Ebine, Makoto Hirai, Miako Sakaguchi, Kazuhide Yahata, Osamu Kaneko, Yumiko Saito-Nakano

**Affiliations:** Department of Parasitology, National Institute of Infectious Diseases, Shinjuku-Ku, Tokyo Japan; Division of Cellular Dynamics, National Institute for Basic Biology, Okazaki, Aichi Japan; Department of Molecular and Cellular Parasitology, Graduate School of Medicine, Juntendo University, Bunkyo-Ku, Tokyo Japan; Department of Parasitology, Graduate School of Medicine, Gunma University, Gunma, Japan; Central Laboratory, Institute of Tropical Medicine (NEKKEN), Nagasaki University, Nagasaki, Nagasaki Japan; Department of Protozoology, Institute of Tropical Medicine (NEKKEN), Nagasaki University, Nagasaki, Nagasaki Japan

**Keywords:** Membrane trafficking, Rab5b GTPase, Myristoylation, Palmitoylation, Adenylate kinase, Parasitophorous vacuole membrane, Tubovesicular network

## Abstract

**Background:**

Rab5 GTPase regulates membrane trafficking between the plasma membrane and endosomes and harbours a conserved C-terminal isoprenyl modification that is necessary for membrane recruitment. *Plasmodium falciparum* encodes three Rab5 isotypes, and one of these, Rab5b (PfRab5b), lacks the C-terminal modification but possesses the N-terminal myristoylation motif. PfRab5b was reported to localize to the parasite periphery. However, the trafficking pathway regulated by PfRab5b is unknown.

**Methods:**

A complementation analysis of Rab5 isotypes was performed in *Plasmodium berghei.* A constitutively active PfRab5b mutant was expressed under the regulation of a ligand-dependent destabilization domain (DD)-tag system in *P. falciparum*. The localization of PfRab5b was evaluated after removing the ligand followed by selective permeabilization of the membrane with different detergents. Furthermore, *P. falciparum* N-terminally myristoylated adenylate kinase 2 (PfAK2) was co-expressed with PfRab5b, and trafficking of PfAK2 to the parasitophorous vacuole membrane was examined by confocal microscopy.

**Results:**

PfRab5b complemented the function of PbRab5b, however, the conventional C-terminally isoprenylated Rab5, PbRab5a or PbRab5c, did not. The constitutively active PfRab5b mutant localized to the cytosol of the parasite and the tubovesicular network (TVN), a region that extends from the parasitophorous vacuole membrane (PVM) in infected red blood cells (iRBCs). By removing the DD-ligand, parasite cytosolic PfRab5b signal disappeared and a punctate structure adjacent to the endoplasmic reticulum (ER) and parasite periphery accumulated. The peripheral PfRab5b was sensitive to extracellular proteolysis after treatment with streptolysin O, which selectively permeabilizes the red blood cell plasma membrane, indicating that PfRab5b localized on the iRBC cytoplasmic face of the TVN. Transport of PfAK2 to the PVM was abrogated by overexpression of PfRab5b, and PfAK2 accumulated in the punctate structure together with PfRab5b.

**Conclusion:**

N-myristoylated *Plasmodium* Rab5b plays a role that is distinct from that of conventional mammalian Rab5 isotypes. PfRab5b localizes to a compartment close to the ER, translocated to the lumen of the organelle, and co-localizes with PfAK2. PfRab5b and PfAK2 are then transported to the TVN, and PfRab5b localizes on the iRBC cytoplasmic face of TVN. These data demonstrate that PfRab5b is transported from the parasite cytosol to TVN together with N-myristoylated PfAK2 via an uncharacterized membrane-trafficking pathway.

**Electronic supplementary material:**

The online version of this article (doi:10.1186/s12936-016-1377-4) contains supplementary material, which is available to authorized users.

## Background

Intracellular malaria parasites secrete hundreds of proteins into their host red blood cell (RBC) cytoplasm, which is necessary for host cell modification and intracellular survival. Many of these exported proteins contain an endoplasmic reticulum (ER) signal sequence and a short pentameric motif called a *Plasmodium* export element (PEXEL) [1]. PEXEL motif-containing proteins are transported through the vesicular transport pathway [2, 3], and might be recognized by a translocon complex located on the parasitophorous vacuole membrane (PVM), *Plasmodium* translocon of exported proteins (PTEX), for the translocation of PEXEL proteins to the cytoplasm of infected red blood cells (iRBCs) [4, 5]. Although translocation of exported proteins that lack a canonical PEXEL motif is also mediated by PTEX, the trafficking mechanism of PEXEL-negative proteins has not been fully elucidated [1, 5].

A membranous extension from the PVM forms a cytoplasmic tubovesicular network (TVN) [6, 7]. TVN can be stained by fluorescent ceramide and have sphingomyelin synthase activity [8]. In addition, a substrate analogue of sphingomyelin synthase inhibits TVN formation and the uptake of nutrients by the parasite [9, 10]. Thus, TVN has been proposed to be a part of sphingolipid-rich rafts and related to nutrient uptake. Several transmembrane proteins and membrane-associated proteins that are post-translationally modified with saturated fatty acids in a process referred to as ‘acylation’ are recruited to the membrane raft domain [11]. Indeed, *Plasmodium falciparum* adenylate kinase 2 (PfAK2), which is modified by two N-terminal acylation sites, i.e., myristoylation and palmitoylation, localizes to the TVN-like membrane protrusion of the PVM [12]. Transport of PfAK2 to the TVN is dependent on its myristoylation and palmitoylation [13], indicating that lipid acylation might be an alternative protein secretion signal to and from the PVM. However, the mechanism of the transport of acylated proteins to iRBC cytoplasm is not clearly understood.

In eukaryotic membrane trafficking, specific membrane fusions and cargo protein sorting are regulated by the Rab family of small guanosine-5′-triphosphatases (GTPases) [14–16]. In conventional Rab GTPases, the isoprenylation of a highly conserved cysteine motif in the carboxyl terminus (C-terminus) is essential for membrane recruitment of Rab [15, 16]. Rab GTPases cycle between two distinct states, a GTP-bound membrane-associated active form and a GDP-bound mainly cytosolic inactive form. A constitutively active mutant, which was created by the introduction of a specific amino acid substitution in the conserved GTP-binding motif, shows reduced GTPase activity [17, 18]. This active mutant stimulates recruitment of specific binding partners on target membrane, and then activates vesicle transport, tethering and fusion [15]. Thus, expression of constitutively active mutant of mammalian Rab5 or Rab8 causes promoting excessive membrane fusion [18, 19]. In case of mammalian Rab6 and yeast Sec4, for which GTPase activity is necessary for completion of their roles, inhibitory effect on membrane fusion is reported [20, 21]. Analyses using an active mutant of Rab provide critical information of its function and subcellular localization in many organisms.

Eleven Rab genes are found in the *Plasmodium* genome [22] and functions have been reported for several members. *Plasmodium* Rab6 and Rab7 are involved in the trafficking from the Golgi and in the endosomal pathways, respectively, during the asexual development in its life cycle [23, 24]. PfRab11a is involved in the post-Golgi trafficking to the invaginated plasma membrane during the daughter merozoite biogenesis in late-stage schizonts [25]. Interestingly, among three *Plasmodium* Rab5 homologues, a conventional Rab5 homologue, PfRab5a, is involved in the haemoglobin uptake at the plasma membrane and the transport to the food vacuole [26]. In contrast, N-terminally myristoylated PfRab5b that lacks a C-terminal cysteine residue localizes to the parasite food vacuole and the plasma membrane [27], indicating functional diversification of Rab5 subfamily members in *Plasmodium*. Apart from this, intracellular malaria parasites secrete hundreds of proteins into the host RBC cytoplasm for which vesicular trafficking is also proposed to be involved. However, very little is known about the role of Rab GTPases in this process.

Functional difference between conventional, C-terminally isoprenylated Rab5 and N-terminally myristoylated Rab5 have been reported in higher plants [28]. Conventional Rab5 regulates endocytic transport in other organisms [29] and also in higher plants [30, 31]. In contrast, *Arabidopsis thaliana* N-terminally myristoylated Rab5 isotype, namely ARA6/RabF1, regulates the transport from the endosome to the plasma membrane [28, 32, 33]. N-terminal myristoylation and palmitoylation are essential for endosome targeting and the function of ARA6/RabF1 [28, 32, 33]. Proteins similar to ARA6/RabF1 are highly conserved among higher plants and regulate plant-specific trafficking pathways [34, 35].

In the present study, the mechanism of N-myristoylated Rab5b-dependent membrane trafficking in *Plasmodium* was clarified. The loss of function of Rab5b was not complemented by other conventional Rab5 isotypes, Rab5a and Rab5c, in *Plasmodium berghei*. In addition, the constitutively active mutant Rab5b in *P. falciparum,* which was expressed under the inducible destabilization domain (DD) system, localized on the iRBC cytoplasmic face of the PVM and the TVM. Furthermore, PfRab5b over-expression prevented the localization of N-acylated PfAK2 on the PVM, and N-acylated PfAK2 was accumulated in the parasite cytosol. This is the first study to show that two N-acylated proteins, PfRab5b and PfAK2, share the same trafficking pathway to PVM in *Plasmodium*.

## Methods

### Ethics statement

All animal procedures were approved by the Institutional Animal Care and Use Committee (No. 212010-2, 213013-2) and conducted at the AAALAC-accredited National Institute of Infectious Diseases, Japan. Human RBCs and plasma were obtained as donations from anonymized individuals at the Japanese Red Cross Society (No. 25J0022).

### Strains and transfection protocols

*Plasmodium berghei* ANKA strain (clone 2.34), contributed by TF McCutchan, was obtained from the Malaria Research and Reference Reagent Resource Center (MR4). Transfection and pyrimethamine selection was performed as described previously [36]. Genomic integration of the plasmid was assessed by polymerase chain reaction (PCR). A complementation assay was performed whether transformed parasites were selected with pyrimethamine after 3 weeks. If a transformant parasite was selected within 3 weeks, genome integration of the plasmid into the PbRab5b locus was assessed by PCR. A strain that expressed PbRab5b-monomeric Azami Green (mAG) (Medical Biological Laboratories) was cloned by limiting-dilution. For analysing parasite growth, 10^4^ parasites were intravenously injected into 5-weeks old BALB/c female mice. *Plasmodium falciparum* line MS822 [37] was cultured as described previously [38], and transfection of *P. falciparum* was performed according to the published method [38, 39]. Briefly, uninfected RBCs were suspended in 400 μl of cytomix containing 100 μg of plasmid DNA. Electroporation was performed in 0.2 cm cuvettes using the BTX Electroporation System (condition: 0.32 kV, 950 μF; Harvard Apparatus, MA, USA). Transfected RBCs were mixed with trophozoite-rich parasite culture at a final concentration of 0.1 % parasitemia. At 3 days post transfection, transfectants were selected with 5 nM WR99210-HCl (a kind gift from D Jacobus) or 2.5 μg/ml of blasticidin S-HCl (BSD; Sigma-Aldrich). Resistant parasites were usually detected before 1 month of culture in the presence of drugs.

### Plasmid construction

pL0006 was obtained from MR4 (deposited by AP Waters) and was used to make a panel of transfection constructs. To replace the genomic sequence encoding PbRab5b, 500 bp upstream and downstream of the *PbRab5b* locus were cloned into *Hind*III–*Pst*I and *Xho*I–*Eco*RI sites of pL0006, respectively (PbRab5b-KO plasmid). A series of open reading frames (ORFs) comprising the sequences encoding PbRab5b, mAG, and the terminator region of *P. berghei* dihydrofolate reductase (PbDT) were PCR-amplified with overlapping oligonucleotides and were then inserted into the *Pst*I site of the PbRab5b-KO plasmid using an In Fusion HD cloning kit (Clontech, USA) to yield PbRab5b-mAG plasmid. For complementation analysis, each gene-of-interest (GOI) was PCR-amplified, and an In Fusion HD cloning kit was used to replace the PbRab5b ORF in the PbRab5b-mAG plasmid.

To construct a plasmid expressing PfRab5b-YFP-DD, a fusion fragment comprising *P. falciparum* chloroquine resistance transporter (CRT) promoter, PfRab5b coding region, yellow fluorescent protein (YFP; from D E Goldberg) and DD domain optimized for C-terminal fusion (Clontech) was PCR-amplified and inserted between attR4 and attR3 sites of pCHD43(II) [40] to yield PfRab5b-YFP-DD plasmid. To construct a plasmid that expresses a RFP-fusion protein, a PfRab5b-YFP-DD fragment and a human dihydrofolate reductase (DHFR) cassette in the PfRab5b-YFP-DD plasmid were replaced with a TagRFP fragment and a BSD-resistance cassette amplified from pLN-ENR-GFP (MR4, deposited by DA Fidock), respectively. PfAK2, PfSec13, PfVPS2, and the apicoplast-targeting signal sequences [41] were PCR-amplified and inserted into the downstream of the CRT promoter in the RFP plasmid. To express the N-terminal 20 amino acids (aa) of PfRab5b fused to YFP-DD, a sequence containing 61–624 bp of the PfRab5b coding region was removed from the PfRab5b-YFP-DD plasmid. In Fusion HD Cloning kit was used to construct all plasmids.

Schematic structures of plasmids are shown in Additional file 1, and all primers are listed in Additional file 2: Table S1 with their sequence.

### Antibodies

Anti-PfTPx-1 and anti-PfTPx-2 were gifts from S-I Kawazu (Obihiro University of Agriculture and Veterinary Medicine) [42, 43]; anti-PfSBP-1 and anti-PfEXP-2 were from T Tsuboi (Ehime University) [44]; and anti-PfBip was from K Kita (University of Tokyo). Rabbit anti-PfEVP1 and anti-PfERD2 antibodies were raised against the previously reported peptides LKFQHDQEFLNYFKRYQDFN and CYFALAKWYGKKLVLPFNGEV [45, 46], respectively, by Operon Biotechnologies (Tokyo, Japan). Anti-GFP antibodies, purchased from Molecular Probes (rabbit anti-GFP antibody, A11122) and Roche (anti-GFP mouse antibody, 11814460001), were used for the indirect immunofluorescence assay and immunoblots to probe YFP, respectively. Antibodies were used for immunoblotting at the following dilutions; anti-PfEXP2 (1:8,000), anti-PfTPx-1 (1:1,000), anti-PfSBP-1 (1:10,000) and anti-GFP (1:100).

### Confocal microscopy

Immunofluorescence assays were performed with the antibodies incubated at the following dilutions; anti-PfTPx-1 (1:200), anti-PfTPx-2 (1:200), anti-PfERD2 (1:100), anti-PfBip (1:200), anti-PfSBP1 (1:5,000), anti-PfEVP1 (1:50), anti-PfEXP2 (1:200), and anti-GFP (1:100). Alexa 488- and Alexa 568-conjugated anti-rabbit IgG, and Alexa 568-conjugated anti-mouse IgG (Molecular Probes) were used as secondary antibodies. Images were acquired using a LSM510 or LSM780 confocal laser-scanning microscope (Zeiss, Germany). To visualize TVN membranes, iRBCs were incubated with 2.5 µM BODIPY Texas Red C_5_-ceramide ((*N*-((4-(4,4-difluoro-5-(2-thienyl)-4-bora-3a,4a-diaza-s-indacene-3-yl)phenoxy)-acetyl)sphingosine, Molecular Probes) for 1–2 h. Samples were washed with RPMI 1640 medium, and the YFP and TR-ceramide fluorescence were captured using an LSM 7-Live laser-scanning microscope (Zeiss).

### Induction and Shld1 washout assay

For the regulated induction of PfRab5b-YFP, parasites transfected with DD-based plasmids were incubated with 0.5 μM Shld1 (Clontech) for the indicated times. After incubation, iRBCs were washed twice with Shld1-free medium, then further incubated with Shld1-free medium for 1 h (Fig. 5) or 2 h (others). Samples were then stained with TR-ceramide for 30 min, and images were acquired using an LSM 7-Live laser-scanning microscope.

### Antibody accessibility assay

iRBCs (1–5 % parasitaemia) were incubated with 0.5 μM Shld1, fixed with 4 % paraformaldehyde in phosphate-buffered saline (PFA-PBS) at 4 °C for 18 h, and permeabilized with 0.01 % saponin-PBS or 0.1 % Triton X-100-PBS for 1 h. Samples were incubated with rabbit anti-GFP antibody and Alexa 568-conjugated anti-rabbit IgG antibody (Molecular Probes).

### Proteinase K accessibility assay

The accessibility by proteinase K was determined as reported previously with some modifications [47]. Briefly, a 5-ml culture of iRBCs (1–5 % parasitaemia) was incubated with 0.5 μM of Shld1 for 24 h, then further incubated with a Shld1-free medium for 2.5 h. RBCs were collected by centrifugation at 500×*g* for 5 min and permeabilized with 500 μl of streptolysin O (SLO) solution (2.5 U/ml SLO in PBS) for 30 min. After SLO treatment, samples were washed twice with 5 ml of PBS, resuspended to 80 µl with PBS, and then aliquoted into two tubes containing 40 µl of each solution. Proteinase K solution (1 µl, 18 mg/ml, Roche 11389200) was added to one tube, and both tubes were incubated on ice for 30 min. Phenylmethylsulfonyl fluoride (PMSF) (5 µl, 200 mM) was added to each sample, followed by 25 µl of 3 × SDS-PAGE buffer, and then samples were incubated at 95 °C for 5 min. Fifteen-microliter samples were loaded into each well of the SDS-PAGE gels.

## Results

### The entire Rab5b GTPase domain is required for the growth of asexual blood stage of *Plasmodium berghei*

**Fig. 1:**
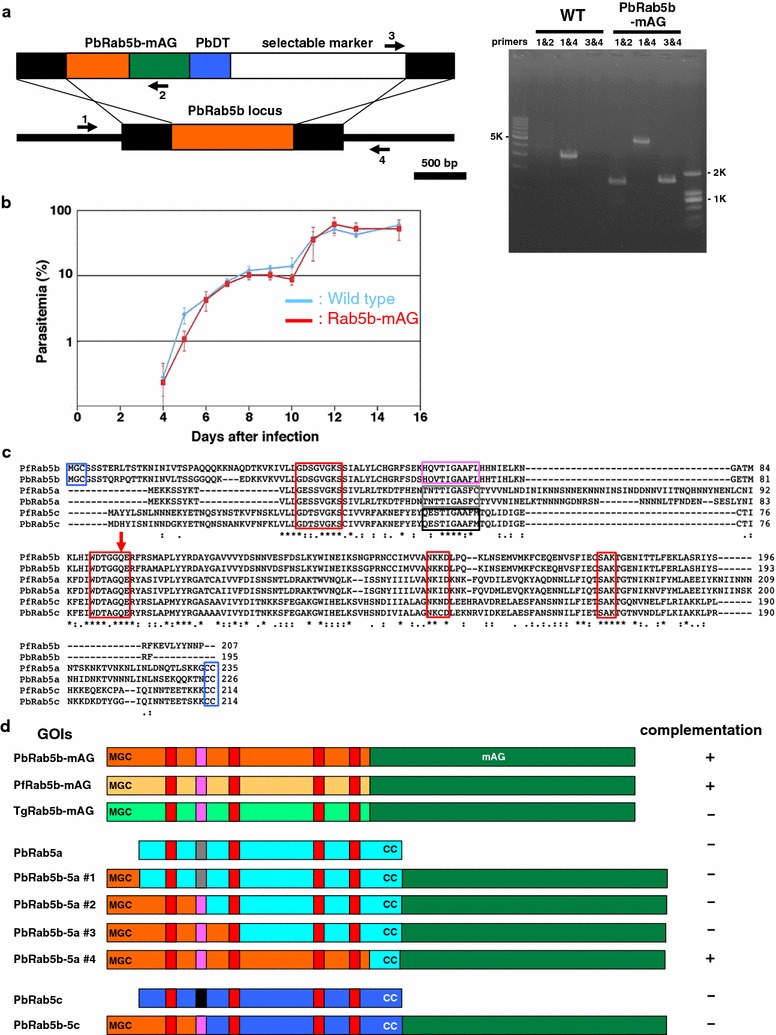
Complementation of *Plasmodium berghei* N-acylated Rab5b with conventional Rab5 and Rab5b from other apicomplexan parasites. **a** Schematic representation of PbRab5b genomic-locus replacement strategy. The targeting construct, consisting of the 5′ and 3′ regions (*two black boxes*) and PbRab5b open reading frame (ORF) (*orange*) fused to mAG (*dark green*), *P. berghei* DHFR 3′ untranslated region (PbDT) (*blue*), and the selectable marker TgDHFR expression cassette (*white*), was integrated into the PbRab5b genomic locus (*orange*) by double-crossover homologous recombination. The positions of the four PCR primers used to confirm the plasmid integration (*right panel*) are indicated. **b** Growth curve of the wild-type parasites (*blue*) and transgenic parasites for which the PbRab5b open reading frame replaced with PbRab5b-mAG (*red*). *Bars* standard deviation (n = 5). **c** Multiple amino acid sequence alignment of *Plasmodium* Rab5 isoforms. The GTP-binding consensus sequences and lipid modification sites are indicated *red* and *blue boxes*, respectively. The effector regions are shown as *grey*, *black* or *magenta boxes*. The conserved glutamine residue which was mutated to create a constitutively active mutant is shown in a *red arrow*. **d** Complementation test of the PbRab5b knockout. The coding region of PbRab5b was replaced with each gene-of-interest (GOI). The result of the complementation analysis is shown at the *right*; +, complemented or −, uncomplemented (n = 3)

Recent attempts to generate a deletion mutant of N-terminally myristoylated and palmitoylated Rab5b in the rodent malaria parasite *P. berghei* (PBANKA_140910) were unsuccessful, suggesting that PbRab5b is essential for the asexual blood stage of the parasite [27]. To further explore the conserved function of Rab5b in apicomplexan parasites, the endogenous PbRab5b locus was replaced with following Rab5 homologues; Rab5b from human malaria pathogen *P. falciparum* (PfRab5b, PF3D7_1310600), Rab5b from the distantly-related apicomplexan parasite *Toxoplasma gondii* (TgRab5b, TGME49_207460), and the other conventional Rab5 isoforms of *P. berghei* (PbRab5a, PBANKA_030800 and PbRab5c PBANKA_020650) (Fig. 1).

First, the genomic locus encoding PbRab5b was replaced with a fragment encoding PbRab5b fused to monomeric Azami Green (mAG) (Fig. 1a). This transgenic parasite grew as well as the wild type (Fig. 1b), indicating that the PbRab5b-mAG fusion protein was functional. The PbRab5b genomic locus was also successfully replaced with PfRab5b fused to mAG (PfRab5b-mAG), indicating that PfRab5b complemented the function of PbRab5b (Fig. 1c, d). This result is supported by the presence of a completely conserved effector sequence (HQVTIGAAFL) between PbRab5b [amino acid residues (aa) 60–69] and PfRab5b (aa 63–72) that specifies the function of Rab proteins [48, 49] (Fig. 1c). In contrast, *P. berghei*-transfected with TgRab5b-mAG, which has a similar effector sequence (aa 66–75, HEVTIGAAFL, with the underline indicating different residue) in addition to a characteristic insertion sequence (aa 165–178, Additional file 3), did not functionally complement PbRab5b (Fig. 1d). These results suggest that the molecular function of Rab5b in *P. berghei* is conserved with *P. falciparum*, but not with *Toxoplasma gondii*.

After drug selection, parasites transfected with plasmids replacing PbRab5b with conventional Rab5 isoforms, PbRab5a and PbRab5c, were not obtained, suggesting Rab5a and Rab5c were unable to complement endogenous PbRab5b (Fig. 1d). To identify the functional regions of PbRab5b, a panel of chimeric constructs, which consisted of PbRab5b with replacements of equivalent regions of PbRab5a or PbRab5c, was expressed. The following chimeric constructs did not complement endogenous PbRab5b; aa 1–34, 1–69, or 1–92 in PbRab5b-mAG proteins were replaced with equivalent regions from PbRab5a (Fig. 1d, PbRab5b–5a #1, #2, and #3, respectively), and aa 1–69 in PbRab5b-mAG was replaced with corresponding sites of PbRab5c (Fig. 1d, PbRab5b–5c). The only chimera that complemented the PbRab5b locus was a construct that included the entire GTPase motif of Rab5b (aa 1–192, PbRab5b–5a #4). These results indicate that PbRab5b functions differently from PbRab5a or PbRab5c, and that the GTPase activity is required for a proper Rab5b function.

### Localization of the constitutively active PfRab5b^Q94L^ mutant in the TVN

Expression of the constitutively active mutant of Rab5 GTPases in mammalian cells, which were recruited to the membrane and accumulated on the target organelle, allows insights into the function and subcellular localization of the transport pathway [18, 50]. In this study, the traffic pathway regulated by *Plasmodium* Rab5b using a constitutively active *Plasmodium* Rab5 mutant was investigated.

At the schizont stage, wild-type PfRab5b localizes near the parasite plasma membrane [27]. The PbRab5b-mAG expressed in the parasite showed a cytosolic pattern (Additional file 4a) over a slightly larger area than the cytosolic marker at the trophozoite stage (Additional file 4b). The constitutively active PbRab5b^Q91L^-mAG mutant (Fig. 1c) accumulated at the periphery of the parasite (Additional file 4c). However, because of the low level of PbRab5b-mAG signal, the precise location of PbRab5b was difficult to determine based on this image.

To examine whether Rab5b was transported to the parasite plasma membrane and then secreted to the PV space, the DD-fused constitutively active PfRab5b^Q94L^ mutant (Fig. 1c) was expressed in *P. falciparum* [51, 52]. In *Toxoplasma,* over-expression of wild-type TgRab5b under the regulation of a DD-tag inhibits egress of daughter tachyzoites from host cells [53]. In contrast, neither PfRab5b-YFP-DD nor PfRab5b^Q94L^-YFP-DD expression altered parasite growth with 2 or 3 days of incubation with Shld1, whereas PfRab5b-YFP-DD protein expression had already plateaued after the 24 h of incubation with Shld1 (Additional file 5), indicating that over-expression of the wild type or constitutively active mutant of PfRab5b did not affect the parasite growth. Another Rab mutant, nucleotide-free PfRab5b^N148I^ mutant, was not successfully expressed by the DD-tag system, thus PfRab5b-YFP-DD and PfRab5b^Q94L^-YFP-DD parasites were used in this analysis.

**Fig. 2:**
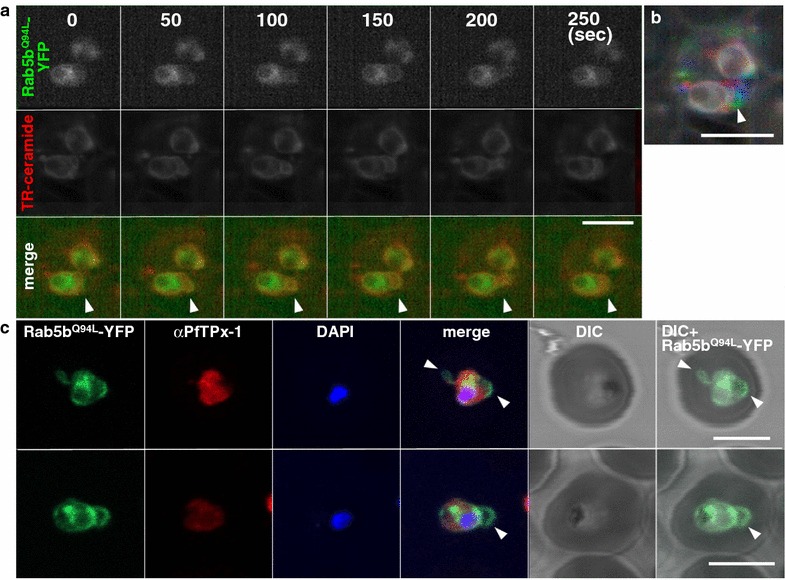
Localization of PfRab5b^Q94L^-YFP-DD to the TVN. **a** Time-lapse imaging of PfRab5b^Q94L^-YFP-DD (*green*) and TR-ceramide (*red*) fluorescence after stabilization with Shld1. PfRab5b^Q94L^-YFP-DD and TR-ceramide was co-localized to a rapidly moving compartment (*arrowheads*). **b** The pseudocolours of TR-ceramide at 0, 100, and 200 s in Fig. 2a were converted to *red*, *green* and *blue*, respectively, and then merged into a single frame. Several extended, mobile TVNs are shown in each colour (*arrowhead* as an example), whereas a stable PVM is shown in *white*. (c) Cells expressing PfRab5b^Q94L^-YFP-DD (*green*) were stained with anti-PfTPx-1 antibody (*red*) and DAPI (*blue*). Cytoplasmic PfTPx-1 was not detected from the TVN, where PfRab5b^Q94L^-YFP-DD (*arrowheads*) localizes. *Bars* 5 µm

First, constitutively active PfRab5b^Q94L^-expressing parasites were double-stained with fluorescent TR-ceramide, which allowed visualization of the PVM and TVN [45]. The PfRab5b^Q94L^-YFP-DD signal was co-localized with TR-ceramide-positive TVN structures after 24 h of incubation with Shld1 (Fig. 2a). PfRab5b^Q94L^-YFP-DD positive TVN loops were mobile, while the PVM was immobile (Fig. 2a, b) the same as recently reported in *P. berghei* [54]. A parasite cytosolic marker, *Plasmodium* 2-Cys peroxiredoxin (PfTPx-1, PF14_0368) [43], was not detected from the PfRab5b^Q94L^-YFP-DD positive TVN loops, indicating that a portion of PfRab5b^Q94L^-YFP-DD localized outside of the parasite (Fig. 2c). These observations suggest that PfRab5b^Q94L^-YFP-DD was secreted from the parasite cytoplasm to the PV space and/or potentially to the RBC-cytoplasmic face of the PVM.

**Fig. 3:**
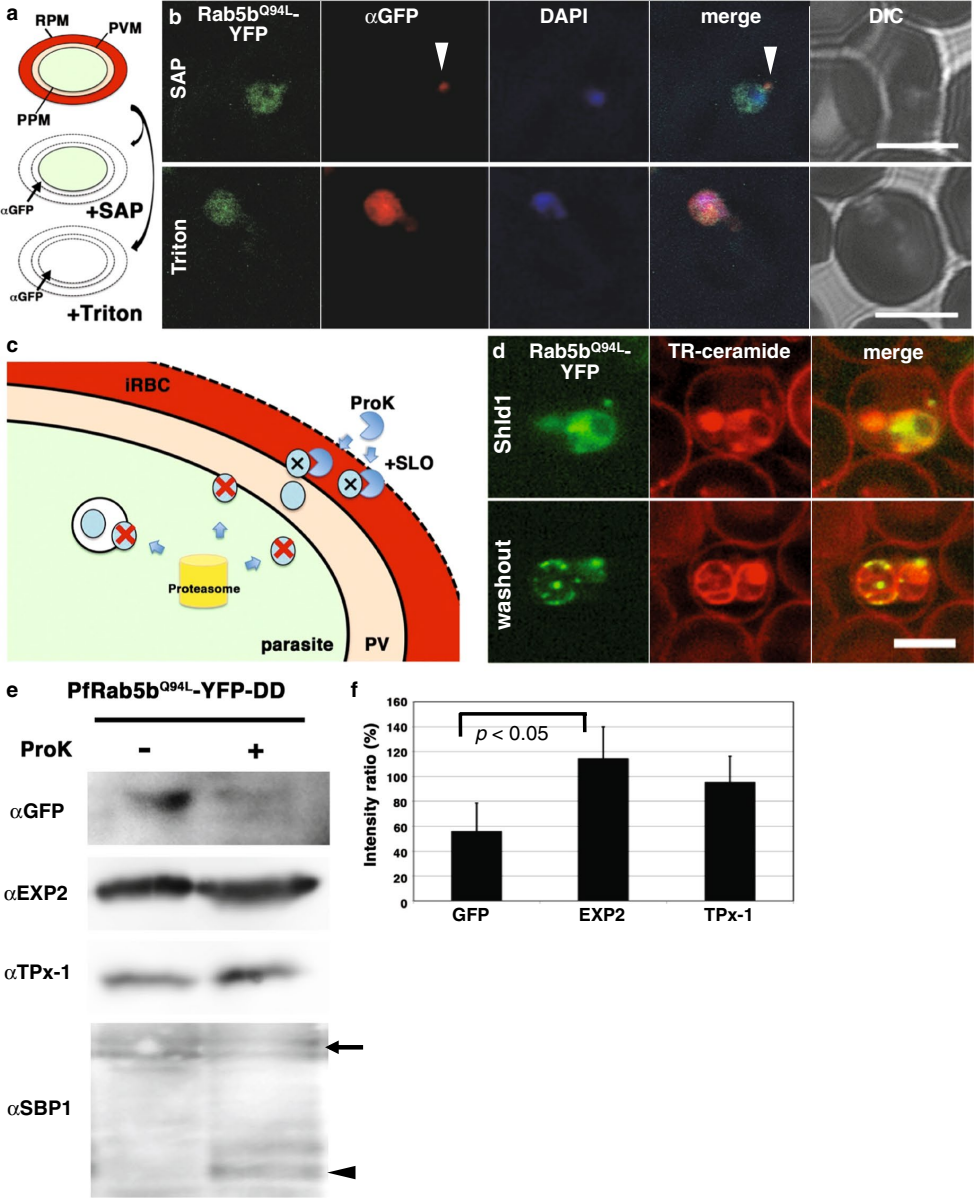
Transport of PfRab5b^Q94L^-YFP-DD to the cytoplasmic face of infected red blood cells on the TVN. **a** The selective permeabilization scheme. Saponin permeabilizes RBC plasma membrane (RPM) and PVM, which allows the detection of proteins localized to the outside of the parasite plasma membrane (PPM) or both sides of the PVM. Triton X-100 permeabilizes RPM, PVM, and PPM, allowing staining of the iRBCs, PV, and parasite cytosol with antibody. **b** Cells expressing PfRab5b^Q94L^-YFP-DD (*green*) were subjected to an antibody accessibility assay with anti-GFP antibody (*red*) prior to permeabilization with saponin (*upper panel*, SAP) or Triton X-100 (*lower panel*, Triton). After the saponin treatment, anti-GFP antibody labelled PfRab5b^Q94L^-YFP-DD secreted to the TVN (*arrowhead*), whereas PfRab5b^Q94L^-YFP-DD in the parasite cytosol was not labelled. Permeabilization with Triton X-100 allowed labelling of both the parasite cytosolic and TVN-localized PfRab5b^Q94L^-YFP-DD with anti-GFP antibody. **c** Schematic of the Shld1 washout assay and the protease accessibility assay. After removal of Shld1, cytosolic DD-tagged proteins are degraded (*red cross* and *blue circle*) by the parasite proteasome (*yellow cylinder*). DD-tagged proteins, inside of intracellular organelles or transported to the outside of the parasite (blue circular), are resistant to the proteasomal degradation. Since streptolysin O (SLO) permeabilizes RPM but not PVM, DD-tagged proteins in the RBC cytoplasm were selectively degraded by extracellular proteinase K (ProK) after permeabilization with SLO (*black cross* and *blue circle*). **d** Sub-cellular localization of PfRab5b^Q94L^-YFP-DD (*green*) after Shld1 stabilization for 24 h (*upper panel*, Shld1) and 2 h after Shld1 washout (*lower panel*, washout). After the removal of Shld1, the punctate signal of PfRab5b^Q94L^-YFP-DD localized to the TR-ceramide (*red*)-labelled parasite periphery. *Bars* 5 µm. **e** Parasites expressing PfRab5b^Q94L^-YFP-DD were subjected to Shld1 washout after stabilization (Fig. 3d), and then permeabilized with SLO before treatment with proteinase K. Anti-PfEXP2 and anti-TPx-1 antibodies were used as a control which is not processed with proteinase K, and a loading control for immunoblot analysis, respectively. Representative image from three independent experiments is shown. **f** The intensity of each band in Fig. 3e were quantitated and the intensity of the band with ProK (+) was divided by that of ProK(−) band. Significance was evaluated by the Student’s *t* test. *Bars* standard deviation (n = 3)

Second, the accessibility of antibodies after permeabilization with different detergents was examined to evaluate the location of constitutively active PfRab5b^Q94L^ proteins. iRBCs were treated with saponin, which permeabilizes the iRBC membrane and the PVM but not the parasite plasma membrane (Fig. 3a, SAP), and then PfRab5b^Q94L^-YFP-DD was stained with anti-GFP antibody [55]. PfRab5b^Q94L^-YFP-DD, transported to outside of parasite, was detected by an anti-GFP antibody after the saponin lysis as punctate structures, only at the peripheral region of the parasite, but not in the parasite cytoplasm, among 20 % of PfRab5b^Q94L^-YFP-DD positive parasites, whereas YFP fluorescence was detected in the cytosol of all PfRab5b^Q94L^-YFP-DD parasite cytosol (Fig. 3b, SAP). These results indicat that secreted PfRab5b^Q94L^-YFP-DD at the periphery was recognized by anti-GFP antibody after treatment with saponin. In contrast, after permeabilization of the parasite plasma membrane with Triton X-100 (Fig. 3a, Triton), parasite cytosolic PfRab5b^Q94L^-YFP-DD was detected with anti-GFP antibody (Fig. 3b, Triton). These results again indicate that a portion of PfRab5b^Q94L^-YFP-DD localized outside of the parasite plasma membrane.

Third, PfRab5b^Q94L^-YFP-DD protein that localizes to the parasite cytosol is considered to be degraded by the parasite proteasome pathway in the absence of Shld1 [51]. Because of the weak proteasome activity in RBCs [56, 57], the effect of host-derived proteasome activity is negligible. Thus, PfRab5b^Q94L^-YFP-DD located inside of intracellular organelles or outside of the parasite cytoplasm would remain intact and YFP fluorescence would be apparent (Fig. 3c) [52]. To evaluate this hypothesis, the alteration of PfRab5b^Q94L^-YFP-DD localization was assessed after a 24-h incubation with Shld1 followed by 2 h of incubation without Shld1. After this procedure, the YFP signal was undetectable in the parasite cytosol, and only punctate structures in the parasite cytoplasm and periphery were detected (16 % of PfRab5b^Q94L^-YFP-DD positive parasites displayed a peripheral YFP signal, n = 42), suggesting that PfRab5b^Q94L^-YFP-DD was compartmentalized in the punctate structure in the cytoplasm and at the periphery of the PVM (Fig. 3d). The Amount of induced protein was decreased to 19 ± 4.1 % after the washout of Shld1 for 2 h compared to protein levels after 24 h incubation with Shld1 (Additional file 5e, f). To further determine whether the peripheral dots of PfRab5b^Q94L^-YFP-DD localized inside of the PV or on the RBC-cytoplasmic face of the PVM, iRBCs were treated with SLO, which permeabilized the iRBC plasma membrane but not the PVM or the parasite plasma membrane [55] (Fig. 3c, SLO), and then treated with a proteolytic enzyme proteinase K. Parasites expressing PfRab5b^Q94L^-YFP-DD were incubated with Shld1 for 24 h, followed by incubation without Shld1 for 2 h (Fig. 3d, washout), and then subjected to immunoblotting after SLO-treatment with or without proteinase K (Fig. 3e). The reduction of the PfRab5b^Q94L^-YFP-DD signal by proteinase K treatment (56.3 ± 22.3 %, n = 3) was significantly greater than that of the control PVM marker, exported protein 2 (PfEXP2) (115 ± 25.3 %, n = 3, *p* < 0.05 between YFP and EXP2) (Fig. 3e, f). As a positive control for degraded proteins, Maurer’s cleft membrane protein, SBP1, was processed from a 48 kDa full length (Fig. 3e, arrow) to 37 kDa luminal domain (Fig. 3e, arrowhead) of Maurer’s cleft [58]. These results indicate that PfRab5b^Q94L^-YFP-DD localized to the surface of the PVM and TVN facing the RBC cytoplasm. Wild-type form of Rab5b construct, PfRab5b-YFP-DD, also showed same response to the Shld1 washout and proteinase K treatment (Additional file 6), indicating that PfRab5b-YFP-DD was also transported to the RBC-cytoplasmic face of PVM/TVN.

### PfRab5b and N-acylated PfAK2 share the same trafficking pathway to the TVN

**Fig. 4:**
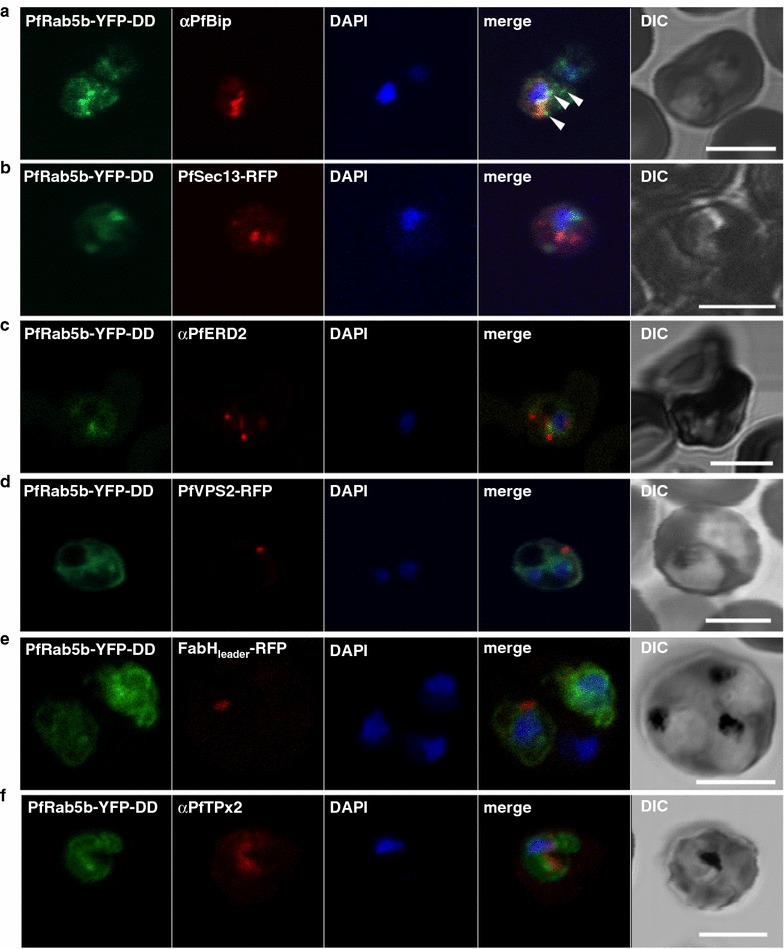
Localization of PfRab5b to a punctate compartment in the parasite cytoplasm. Triple staining with PfRab5b-YFP-DD (*green*), DAPI (*blue*) and one of the following markers (*red*): PfBip (**a**, ER), PfSec13-RFP (**b**, ER exit site), PfERD2 (**c**, Golgi), PfVPS2-RFP (**d**, putative multivesicular body/endosome), FabH_leader_-RFP (**e**, apicoplast), or PfTPx-2 (**f**, mitochondria) after 24 h incubation with Shld1. PfRab5b-YFP-DD localized adjacent to the Bip signal (*arrowheads*). *Bars* 5 µm

To determine the localization of PfRab5b within the parasite, the transgenic parasite line, PfRab5b-YFP-DD, was double-stained with organelle marker proteins; PfBip (ER) [46], PfSec13 (ER exit site) [23], PfERD2 (Golgi) [46], PfVPS2 (putative multivesicular body/endosome) [59, 60], FabH_leader_-RFP (apicoplast) [41], and PfTPx-2 (mitochondria) [42]. Although PfRab5b-YFP-DD staining partially overlapped with PfBip staining, punctate YFP-positive structures localized adjacent to the PfBip signal (Fig. 4a, arrowheads). All other marker proteins did not co-localize with PfRab5b-YFP-DD (Fig. 4b–f). These data suggest that PfRab5b associated with a compartment close to the ER, presumably at the initial step of membrane trafficking.

**Fig. 5:**
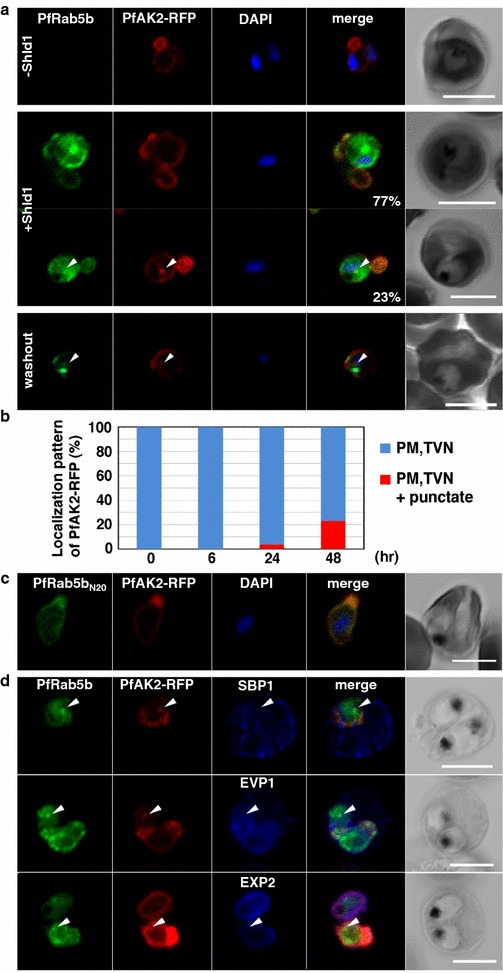
PfRab5b expression disturbed the transport of PfAK2 to the TVN. **a** In the absence of Shld1, PfAK2-RFP (*red*) localized to the TVN, while PfRab5b-YFP-DD signal was not detect (−Shld1, *upper panel*). After Shld1 stabilization for 48 h, PfRab5b-YFP-DD (*green*) and PfAK2-RFP co-localized in the TVN-like structures (+Shld1, *middle-upper panel*). In 77 % of PfRab5b-YFP-DD-positive parasites, PfAK2-RFP localized to the TVN, while in 23 % of PfRab5b-YFP-DD positive parasites, PfAK2-RFP signals also accumulated in the punctate compartment within the parasite (+Shld1, *middle-lower panel, arrowhead*). Co-localization of PfRab5b-YFP-DD and PfAK2-RFP at the punctate compartment remained even after removal of Shld1 (washout, *lower panel*, *arrowhead*). **b** The *bar graph* showing the localization pattern of PfAK2-RFP at 0, 6, 24, and 48 h after Shld1 treatment. **c** No punctate localization of PfAK2-RFP was apparent with co-expression of PfRab5b_N20_-YFP-DD. **d** Effect of PfRab5b over-expression on the exported proteins PfSBP1, PfEVP1, and PfEXP2. In contrast to PfAK2-RFP, these proteins did not accumulate in the PfRab5b- and PfAK2-double positive punctate compartments (*arrowhead*)

Adenylate kinase 2 (PfAK2, PF08_0062) is an N-terminally myristoylated and palmitoylated protein that is transported to the PVN and TVN [12, 13]. PfAK2 does not possess a canonical PEXEL motif and N-terminal acylation is essential for PfAK2 to pass through the parasite plasma membrane [13]. The N-terminally myristoylated and palmitoylated modification was also essential for the membrane association of PfRab5b [27], and transport to the TVM (Additional file 6). In mammalian cells, over-expression of Rab5 proteins stimulated aggregation on the membrane and affected trafficking of the cargo protein [18, 61]. To test whether PfAK2 shares the same trafficking pathway with PfRab5b, full-length PfAK2 fused to RFP (PfAK2-RFP) was co-expressed with PfRab5b-YFP-DD. In the absence of Shld1, PfRab5b-YFP signal was not detected at all, and PfAK2-RFP localized at the parasite periphery and TVN (Fig. 5a, −Shld1). After 48 h of incubation with Shld1, PfAK2-RFP signal was detected at the parasite periphery and the TVN in 77 % of PfRab5b-YFP-positive parasites. In addition, a punctate profile, which appeared to be aggregated PfRab5b-YFP and PfAK2-RFP signals, was found in the parasite cytoplasm in 23 % of PfRab5b-YFP-positive parasites, suggesting that PfRab5b-YFP-DD and PfAK2-RFP were compartmentalized in the same punctate structure (Fig. 5a, middle, arrowheads in +Shld1). Compartmentalized PfAK2-RFP was still detectable even after 1 h of Shld1 washout (Fig. 5a, lower, washout). The ratio of the compartmentalized PfAK2-RFP increased with time after exposure to Shld1 [0 h: 0 % (n = 18), 6 h: 0 % (n = 15), 24 h: 3.7% (n = 27), and 48 h: 23.1 % (n = 26)] (Fig. 5b). When PfAK2-RFP was co-expressed with the YFP-DD protein fused to the N-terminal 20 aa of PfRab5b (PfRab5b_N20_-YFP-DD), which lacked the functional GTPase domain, the PfAK2-RFP positive punctate was not detected in the parasite cytoplasm (Fig. 5c), indicating that the GTPase activity of PfRab5b is required for the accumulation of PfAK2-RFP in vesicles or membranous structure within the parasite.

These data suggest that the over-expression of functional PfRab5b increased PfRab5b-mediated membrane fusion, which, in turn, induced the aggregation of N-acylated PfAK2. To determine whether PfRab5b also participates in the trafficking of other exported proteins, the localization of non-N-acylated proteins, PfEVP1 (a predicted PEXEL-positive transmembrane protein), PfSBP1 (a PEXEL-negative transmembrane protein), and the PVM marker protein PfEXP2, were also examined. In contrast to PfAK2-RFP, these proteins did not accumulate in the PfRab5b-YFP-DD and PfAK2-RFP-positive compartments within the parasite (Fig. 5d). The result suggest that their export to the iRBC cytoplasm was dependent on PfRab5b, and that PfRab5b plays a specific role in the export of N-acylated proteins to the TVN.

## Discussion

Here, this study showed that Rab5b was essential for *Plasmodium* growth and that Rab5a and Rab5c did not complement Rab5b in *P. berghei*, confirming a previous report [27]. The constitutively active PfRab5b mutant was secreted into the PVM and TVN in iRBCs and localized on the RBC-cytoplasmic face of these membranes. Induced over-expression of PfRab5b by DD system resulted in the accumulation of N-acylated PfAK2 in the PfRab5b-positive compartment within the parasite, suggesting that the PfAK2 shares a trafficking pathway which is Rab5b dependent. Analysis of the transport of other N-acylated proteins, such as PfCDPK1 [62, 63], will be required to further define the Rab5b-dependent pathway. *Plasmodium falciparum* secretes hundreds of proteins into RBC cytoplasm, most of which are transported through the PVM-localized translocon PTEX [1, 4]. In contrast to PfAK2, the transport of non-N-acylated proteins, EVP1 and SBP1, which are expected to utilize PTEX, was not perturbed by the over-expression of PfRab5b. These results suggest the presence of a specific trafficking pathway for N-acylated proteins and Rab5b.

### Hypothetical model of the transport of PfRab5b from parasites to the TVN

**Fig. 6:**
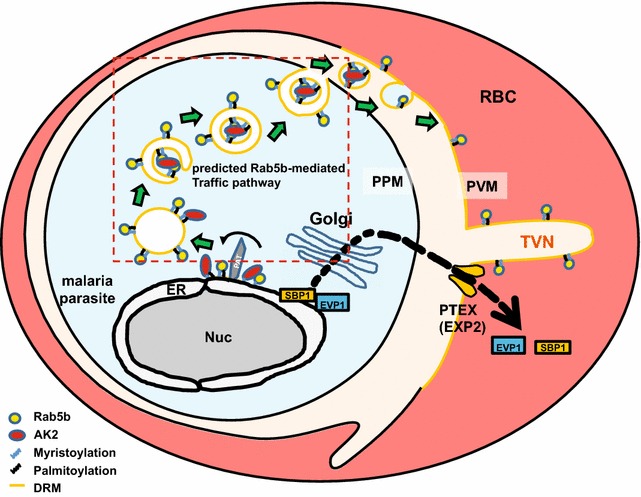
The hypothetical model of trafficking pathway involved in PfRab5b. N-acylated proteins, such as PfAK2, are recruited to novel compartment(s) adjacent to the ER, and packed into the multivesicular body or an autophagosome-like double membrane structure together with PfRab5b. The multivesicular body fuses with the PPM, and internal vesicles fuses with the PVM. Rab5b is clustered on the TVN. Nuc, nucleus; RBC, red blood cell; PTEX, *Plasmodium* translocon of exported proteins; PAT, palmitoyltransferase. EVP1 and SBP1 are exported proteins with and without the *Plasmodium* export element (PEXEL) signal, respectively

Although not all steps are experimentally validated, a proposed model consistent with this observations of PfRab5b-mediated transport of N-acylated proteins from the parasite to the RBC cytoplasm was shown (Fig. 6). The elements of the model are as follows: Step 1: N-acylated proteins, including PfRab5b and PfAK2, are recruited to a novel compartment, either close to or within the ER, where they are anchored tightly to the membrane after their N-terminal Gly and Cys residues are myristoylated and palmitoylated, respectively. The existence of this step is supported by the adjacent localizations of PfRab5b-YFP-DD and the ER marker PfBip (Fig. 4a). Step 2: N-acylated proteins on the vesicles that are released from the ER generate a multivesicular body (MVB), which lacks VPS2 or an autophagosome-like double membrane structure together with PfRab5b, and N-acylated cargo proteins are internalized in the internal vesicles. The existence of this step is supported by the presence of residual PfRab5b-YFP-DD within the parasite after the removal of Shld1 (Figs. 3d, 5a). Over-expression of PfRab5b induced the accumulation of PfAK2 in this compartment (Fig. 5a), suggesting that PfRab5b positively regulates this step (Fig. 6, red box). Step 3: The PfRab5b-positive vesicle fuses with the parasite plasma membrane in a PfRab5b-dependent manner and releases internal vesicles that contain PfRab5b and cargo proteins into the PV space. This step is proposed based on the mechanism for ARA6/RabF1-dependent vesicle trafficking in *Arabidopsis* [32]. Step 4: Cargo proteins released into the PV space fuse with the PVM and supply membrane components to newly generated PVM and TVN in iRBCs, and N-acylated proteins enter the iRBC cytoplasm. This process is supported by the detection of PfRab5b on the RBC cytoplasmic face of the PVM- and TVN (Figs. 2, 3). Step 5: PVM lipid compositions might be heterogeneous and form a specific lipid domain within the PVM where N-acylated proteins cluster (Fig. 6) [12]. Such membrane domain is consistent with a reported detergent-resistant membrane fraction where palmitoylated proteins accumulate [12, 64, 65].

### The palmitoylation motif is required for the PfRab5b transport to the RBC cytoplasm

When myristoylation or palmitoylation sites at Gly and Cys, respectively, were disrupted, both membrane recruitment [27] and secretion of PfRab5b to the PV (Additional file 6) were inhibited, indicating that either myristoylation or palmitoylation are required for transport of PfRab5b beyond the parasite plasma membrane. Specifically, the partial patch-like accumulation of PfRab5b^C3A^-YFP-DD in the parasite cytoplasm after the removal of Shld1 resembles that of the PfAK2^C4A^ mutant, in which the palmitoylation site is altered [13]. The membrane structures where PfRab5^C3A^-YFP-DD and PfAK2^C4A^ accumulate might be novel compartments where N-acylated proteins are selectively recognized and packed. Contiguous localization of PfRab5b-YFP-DD with an ER marker suggests that palmitoylation of Rab5b occurs in the proximal region of the ER (Fig. 4). This result is consistent with the finding in plants that the disruption of the palmitoylation site in ARA6/RabF1 resulted in the change of its localization from endosomes to the ER [28].

Palmitoylation of proteins is regulated by a family of Asp-His-His-Cys (DHHC) motif-containing multi-transmembrane proteins, and *P. falciparum* and *Toxoplasma gondii* possess 12 and 18 members of this protein family, respectively [66, 67]. Each protein with a DHHC motif exhibits a distinct localization and target protein specificity, therefore, an ER-localized member is a prime candidate for palmitoylation of PfRab5b and cargo proteins in this pathway. PbDHHC5 and PbDHHC8 are good candidates because they localize to a compartment close to the ER and unidentified vesicles, respectively [67]. Although it is tempting to speculate that a palmitoylation-depalmitoylation cycle may occur in the iRBC cytoplasm to facilitate vesicle trafficking of the parasite in the iRBC cytoplasm, further studies are required to assess this possibility.

### Accumulation of PfRab5b on the PVM and TVM

Clustering of N-acylated proteins, including PfRab5b, within the TVN (Figs. 2c, 3b) indicates the presence of a specific membrane sub-domain stained with TR-ceramide in the TVN. The cholesterol-rich, detergent-resistant, membrane domain (DRM) is a candidate membrane sub-domain where N-acylated proteins are preferentially localized in other organisms [11, 64]. PTEX is reported to form dot-like structures on the PVM, indicating the presence of hotspots of protein translocation to the RBC cytoplasm in *P. falciparum* [68] and in *P. berghei* [54] and PTEX components were identified in the DRM fraction from *P. falciparum* iRBCs [69]. However, PfAK2 and PfRab5b were not detected in this DRM fraction by the same analysis [69]. The identification of the extracellular membrane domain where PfAK2 and PfRab5b were detected needs further investigation. Identification of Rab5b effector proteins would stimulate the understanding of this PfRab5b-regulated trafficking pathway.

### Evolution of the PfRab5b-dependent transport pathway

From an evolutionary perspective, the Rab5 family is divided into Rab5 and Rab22 subgroups [70]. The divergence of the Rab5 and Rab22 subgroups likely occurred early in the evolution of eukaryotes, and Rab5 and Rab22 sub-groups regulate different transport pathways in mammalian cells [71]. Both subgroups are widely conserved among eukaryotes, including land plants and *Apicomplexa* [70]. In plants and *Apicomplexa*, conventional C-terminally isoprenylated Rab5 and N-terminal acylated Rab5 belong to the Rab5 and Rab22 sub-groups, respectively [70]. These findings support the conclusion that the N-terminal acylated Rab5 family, e.g., plant ARA6/RabF1 and apicomplexan Rab5b, mediates transport pathways that are distinct from those of conventional Rab5 [32, 33, 53]. Together with the data provided in this study, it is reasonable to conclude that apicomplexan Rab5b was evolved to regulate protein transport to the PVM. Consistent with this idea, *Babesia* and *Theileria,* other apicomplexan parasite closely related to *Plasmodium,* possess neither PV nor Rab5b [72, 73]. The observation that TgRab5b did not complement PbRab5b function (Fig. 1d), may suggest species-specific interaction of Rab5b with its effector proteins, which needs future investigation. Furthermore, Rab5b-type Rab GTPases are conserved in the related Alveolata protozoan *Perkinsus* [34, 70]. Further analysis of Rab5b in other living organisms, such as *Toxoplasma*, more distantly related protozoa that belongs to Superphylum *Alveolata* (such as *Perkinsus*) and plants will illuminate the importance of PfRab5b in the evolution of membrane trafficking.

## Conclusions

N-myristoylated *Plasmodium* Rab5b plays a role that is distinct from that of conventional C-terminal isoprenylaed Rab5 isotypes, Rab5a and Rab5c. PfRab5b localizes to a compartment close to the ER, translocated to the lumen of the organelle, and co-localizes with N-myristoylated protein, PfAK2. PfRab5b and PfAK2 are then transported to the TVN, and PfRab5b localizes on the iRBC cytoplasmic face of TVN. These data demonstrate that N-myristoylated proteins, PfRab5b and PfAK2, share the uncharacterized trafficking pathway to PVM.

## Additional files

10.1186/s12936-016-1377-4 Structures of constructs used in this study. Plasmids used for the analysis of *P. berghei* (a) or *P. falciparum* (b). For coexpression of PbRab5b-mAG and RFP, a fusion fragment comprising the promoter region of the gene encoding the *P. falciparum* chloroquine resistance transporter (CRT) promoter [1], TagRFP amplified from the pTagRFP-C plasmid (Evrogen), and PbDT were PCR-amplified using overlapping oligonucleotides, and the In Fusion HD cloning kit was used to insert the DNA fragment into the XhoI site of the PbRab5b-mAG plasmid (PbRab5b-mAG+RFP). For single crossover transfection of constitutive active PbRab5b^Q91L^ mutant, an upstream sequence encompassing nucleotide positions at –1,500 bp to –500 bp of PbRab5b was inserted into HindIII site of the plasmid for double crossover (PbRab5b^Q91L^-mAG for single crossover). Construction of PbRab5b-mAG, PbRab5b Chimeric, GOI-YFP-DD, RFP, GOI-RFP was described in the Materials section of the main manuscript.Reference[1] van Dooren GG, Marti M, Tonkin CJ, Stimmler LM, Cowman AF, McFadden GI. (2005) Development of the endoplasmic reticulum, mitochondrion and apicoplast during the asexual life cycle of *Plasmodium falciparum*. Mol Microbiol; 57:405-19.
10.1186/s12936-016-1377-4 List of oligonucleotide sequences used for plasmid construction.
10.1186/s12936-016-1377-4 Multiple-alignment of amino acids sequences of *Plasmodium* Rab5b and *Toxoplasma* Rab5b. The GTP-binding box (red boxes) and the effector domain (light green box) are shown. Amino acids in the blue box indicate N-terminal myristoyl and palmitoyl modification sites. *Toxoplasma gondii* Rab5b possesses atypical insertion sequences at amino acid positions 165-182, which are not present in *Plasmodium* Rab5b.
10.1186/s12936-016-1377-4 Peripheral localization of PbRab5b-mAG in trophozoite stage of parasites. (a) Fluorescence image of PbRab5b-mAG in trophozoite-stage parasites. Transgenic parasites expressing PbRab5b-mAG under the regulation of PbRab5b promoter were fixed with PFA, and faint cytosolic fluorescence of mAG were detected (green). Nuclei were stained with DAPI (blue). In control wild-type parasites, mAG fluorescence signal was not detected. Bar, 10 μm. (b) Magnified images of trophozoites expressing both PbRab5b-mAG and TagRFP. Trophozoite stage of parasites expressing PbRab5b-mAG (green) and cytosolic TagRFP (red) were fixed and the mAG and TagRFP fluorescence signals were obtained. Histograms of the green and red intensities along the white arrow are shown in the right graph. Black arrowheads indicate regions where stronger PbRab5b-mAG signal were detected compared to the TagRFP signal. Bar, 5 μm. (c) Peripheral localization of PbRab5b^Q91L^-mAG expressed under the regulation of PbRab5b promoter in trophozoite-stage parasites. A constitutively active PbRab5b^Q91L^-mAG mutant, in which Gln at aa 91 was replaced with Leu [1,2] was integrated into the upstream of PbRab5b genomic locus by single crossover method. Fluorescence of PbRab5b^Q91L^-mAG mutant protein was accumulated at the periphery of the parasite (green). Nuclei were stained with DAPI (blue). Bar, 5 μm.References[1] Li G, Barbieri MA, Colombo MI, Stahl PD (1994) Structural features of the GTP-binding defective Rab5 mutants required for their inhibitory activity on endocytosis. J Biol Chem 269: 14631-14635.[2] Stenmark H, Parton RG, Steele-Mortimer O, Lutcke A, Gruenberg J, et al. (1994) Inhibition of rab5 GTPase activity stimulates membrane fusion in endocytosis. EMBO J 13: 1287-1296.
10.1186/s12936-016-1377-4 No growth defect by the expression of PfRab5b-YFP-DD or PfRab5b^Q94L^-YFP-DD seen in the growth curve. Growth curve of parasites expressing PfRab5b-YFP-DD (a) or PfRab5b^Q94L^-YFP-DD (b). Parasitemia (%) was measured after treatment with (green and yellow) or without (blue and red) 0.5 μM Shld1 in the presence of 5 nM WR99210-HCl. (c) Immunoblot using anti-GFP antibody to probe PfRab5-YFP-DD showed that PfRab5-YFP-DD was highly expressed after the incubation with 0.5 μM of Shld1 for 24 h. Tpx1 is a loading control. (d) Quantification of intensity of PfRab5-YFP-DD in Additional Figure 5c. (e) Immunoblot of PfRab5-YFP-DD before or after washout of Shld1 for 2 h. (f) Quantification of intensity of PfRab5-YFP-DD in Additional Figure 5e.
10.1186/s12936-016-1377-4 N-terminal myristoyl and palmitoyl modification of PfRab5b is essential for its transport to the TVN. (a) Subcellular localization of wild-type and mutated PfRab5b-YFP-DD (green) after 24-h Shld1 stabilization (left, Shld1) and after following 2-h Shld1 washout (right, washout). The TR-ceramide red fluorescent dye (red) labels the parasites and iRBC membranes. Washing out of Shld1 resulted in the loss of GFP signals from G2A and C3A mutants. (b) Immunoblotting after proteinase K (ProK) treatment of iRBCs permeabilized with streptolysin O. Anti-PfEXP2 antibody is used as a control which is not processed with proteinase K.

## Abbreviations

PEXEL: *Plasmodium* export element
PVM: parasitophorous vacuole membrane
PTEX: *Plasmodium* translocon of exported proteins
PVM: parasitophorous vacuole membrane
TVN: tubovesicular network
PfAK2: *Plasmodium falciparum* adenylate kinase 2
GTPases: guanosine-5′-triphosphatases
DD: destabilization domain
mAG: Monomeric Azami Green
ORFs: open reading frames
PbDT: *Plasmodium berghei* dihydrofolate reductase
GOI: gene-of-interest
SLO: streptolysin O
CRT: *Plasmodium falciparum* chloroquine resistance transporter
DHFR: dihydrofolate reductase
PFA: paraformaldehyde
PBS: phosphate-buffered saline
PMSF: phenylmethylsulfonyl fluoride
BSD: blasticidin S
