# Supplementary material for: Plasmodium Rab5b is secreted to the cytoplasmic face of the tubovesicular network in infected red blood cells together with N-acylated adenylate kinase 2
Source: Malar J. 2016 Jun 17;15:323. doi: 10.1186/s12936-016-1377-4 (PMC4912828; doi:10.1186/s12936-016-1377-4)
Supplement: Supplementary file 1 — 10.1186/s12936-016-1377-4 Structures of constructs used in this study. Plasmids used for the analysis of P. berghei (a) or P. falciparum (b). For coexpression of PbRab5b-mAG and RFP, a fusion fragment comprising the promoter region of the gene encoding the P. falciparum chloroquine resistance transporter (CRT) promoter [1], TagRFP amplified from the pTagRFP-C plasmid (Evrogen), and PbDT were PCR-amplified using overlapping oligonucleotides, and the In Fusion HD cloning kit was used to insert the DNA fragment into the XhoI site of the PbRab5b-mAG plasmid (PbRab5b-mAG+RFP). For single crossover transfection of constitutive active PbRab5bQ91L mutant, an upstream sequence encompassing nucleotide positions at –1,500 bp to –500 bp of PbRab5b was inserted into HindIII site of the plasmid for double crossover (PbRab5bQ91L-mAG for single crossover). Construction of PbRab5b-mAG, PbRab5b Chimeric, GOI-YFP-DD, RFP, GOI-RFP was described in the Materials section of the main manuscript.Reference[1] van Dooren GG, Marti M, Tonkin CJ, Stimmler LM, Cowman AF, McFadden GI. (2005) Development of the endoplasmic reticulum, mitochondrion and apicoplast during the asexual life cycle of Plasmodium falciparum. Mol Microbiol; 57:405-19. [file 12936_2016_1377_MOESM1_ESM.pdf]

Figure S1

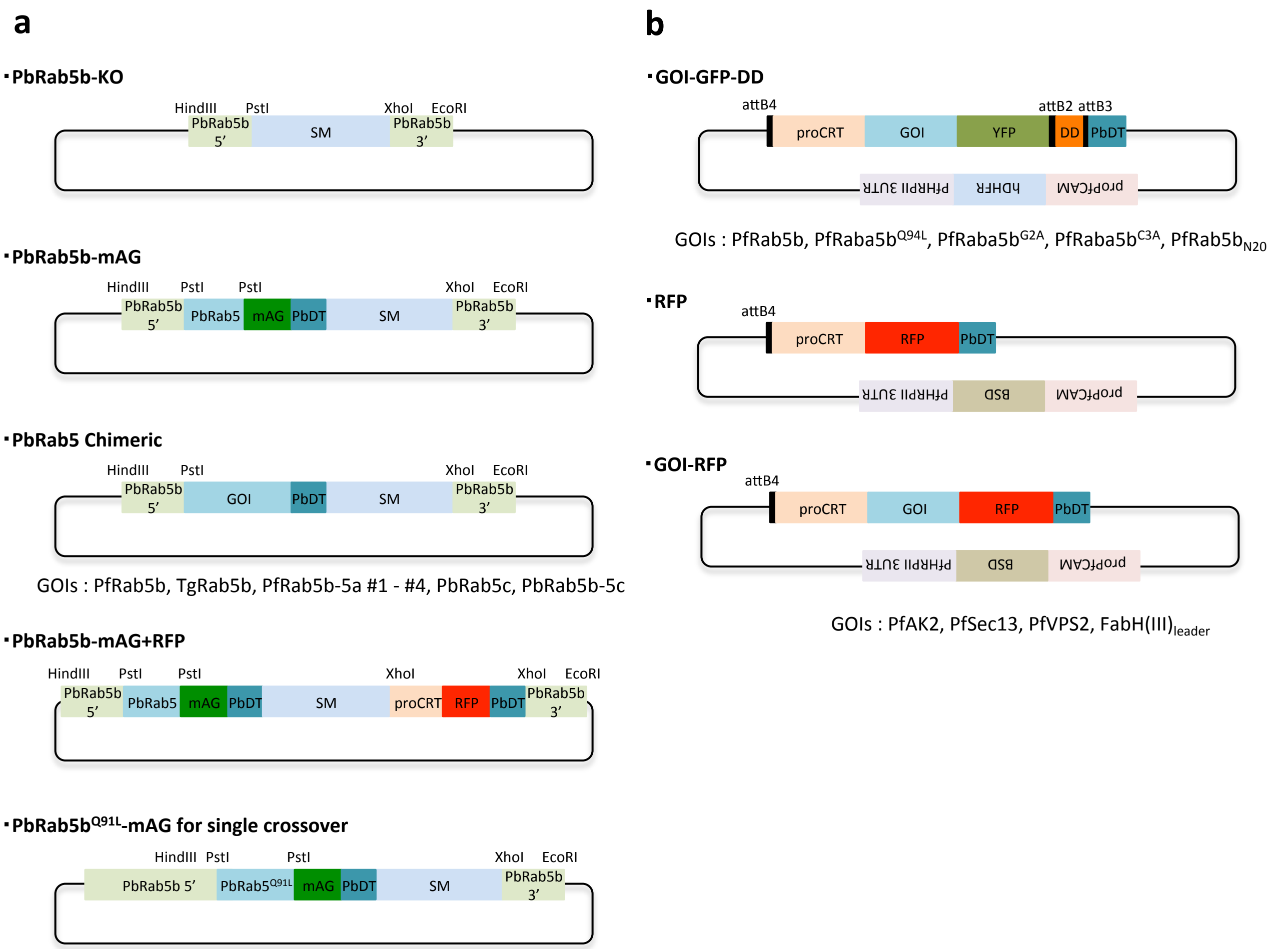

a
・PbRab5b-KO
HindIII PstI XhoI EcoRI
PbRab5b 5′
SM
PbRab5b 3′
・PbRab5b-mAG
HindIII PstI PstI XhoI EcoRI
PbRab5b 5′
PbRab5
mAG
PbDT
SM
PbRab5b 3′
・PbRab5 Chimeric
HindIII PstI XhoI EcoRI
PbRab5b 5′
GOI
PbDT
SM
PbRab5b 3′
GOIs : PfRab5b, TgRab5b, PfRab5b-5a #1 - #4, PbRab5c, PbRab5b-5c
・PbRab5b-mAG+RFP
HindIII PstI PstI XhoI XhoI EcoRI
PbRab5b 5′
PbRab5
mAG
PbDT
SM
proCRT
RFP
PbDT
PbRab5b 3′
・PbRab5b^{Q91L}-mAG for single crossover
HindIII PstI PstI XhoI EcoRI
PbRab5b 5′
PbRab5^{Q91L}
mAG
PbDT
SM
PbRab5b 3′
b
・GOI-GFP-DD
attB4 attB2 attB3
proCRT
GOI
YFP
DD
PbDT
PfHRPII 3UTR
hDHFR
proPfCAM
GOIs : PfRab5b, PfRaba5b^{Q94L}, PfRaba5b^{G2A}, PfRaba5b^{C3A}, PfRab5b$_{N20}$
・RFP
attB4
proCRT
RFP
PbDT
PfHRPII 3UTR
BSD
proPfCAM
・GOI-RFP
attB4
proCRT
GOI
RFP
PbDT
PfHRPII 3UTR
BSD
proPfCAM
GOIs : PfAK2, PfSec13, PfVPS2, FabH(III)$_{leader}$
