# Supplementary material for: Plasmodium Rab5b is secreted to the cytoplasmic face of the tubovesicular network in infected red blood cells together with N-acylated adenylate kinase 2
Source: Malar J. 2016 Jun 17;15:323. doi: 10.1186/s12936-016-1377-4 (PMC4912828; doi:10.1186/s12936-016-1377-4)
Supplement: Supplementary file 3 — 10.1186/s12936-016-1377-4 Multiple-alignment of amino acids sequences of Plasmodium Rab5b and Toxoplasma Rab5b. The GTP-binding box (red boxes) and the effector domain (light green box) are shown. Amino acids in the blue box indicate N-terminal myristoyl and palmitoyl modification sites. Toxoplasma gondii Rab5b possesses atypical insertion sequences at amino acid positions 165-182, which are not present in Plasmodium Rab5b. [file 12936_2016_1377_MOESM3_ESM.pdf]

Figure S2

```
PbRab5b   MGCGSSTQRPQTTKNINVLTSSGGQQK------EDKKVKVVLLGDSGVGKSSIALYLCHGRFSDSHQVTIGAAFLHHTIHLKN-GETMKLHIWDTGGQERFRAMAPLYYRDA  105
PfRab5b   MGCSSSTERLTSTKNINIVTSPAQQQKK---NAQDTKVKIVLLGDSGVGKSSIALYLCHGRFSEKHQVTIGAAFLHHNIELKN-GATMKLHIWDTGGQERFRSMAPLYYRDA  108
TgRab5b   MGCTASSTASAGGESQLRMTNAGGSLDDGFSERLNLEAKIVLLGDSGVGKSSLALRFCRGRFPQYHEVTIGAAFLQQTIRVGDDGSQLKLYIWDTGGQERFRAMAPLYYRDA  112
          *** :*:      :.   :*... . .       : :.*:************:** :*:***.: *:********::.*.: :  *  :**:***********:*********

PbRab5b   YGAIVVYDSNNVDSFNSLKYWINEIKSSGPRNCCIMVVANKKD-LPQKINSE------------------MVMKFCKEQHVSFIECSAKTGENIKTLFERLASHIYSRFZ--  196
PfRab5b   YGAVVVYDSNNVESFDSLKYWINEIKSNGPRNCCIMVVANKKD-LPQKLNSE------------------MVMKFCEQENVSFIECSAKTGENITTLFEKLASRIYSRFKEV  201
TgRab5b   AGAVVVYDVTNPASMDAVRFWVEELKQRGPANCCIAVAANKSDSMENSENAEPPPEEGTAGVDVEAERRAEMKKYCAAEGMLFVECSAKTGCNVGLLFEQLAKEVFEQLKQS  224
           **:**** .*  *::::::*::*:*. ** **** *.***.* : :. *:*                   : *:*  : : *:******* *:  ***:**..::.::

PbRab5b   -------  196
PfRab5b   LYYNNPZ  208
TgRab5b   MMEL---  228
```
