# Supplementary figures and images for: Plasmodium Rab5b is secreted to the cytoplasmic face of the tubovesicular network in infected red blood cells together with N-acylated adenylate kinase 2
Source: Malar J. 2016 Jun 17;15:323. doi: 10.1186/s12936-016-1377-4 (PMC4912828; doi:10.1186/s12936-016-1377-4)

Figure S3

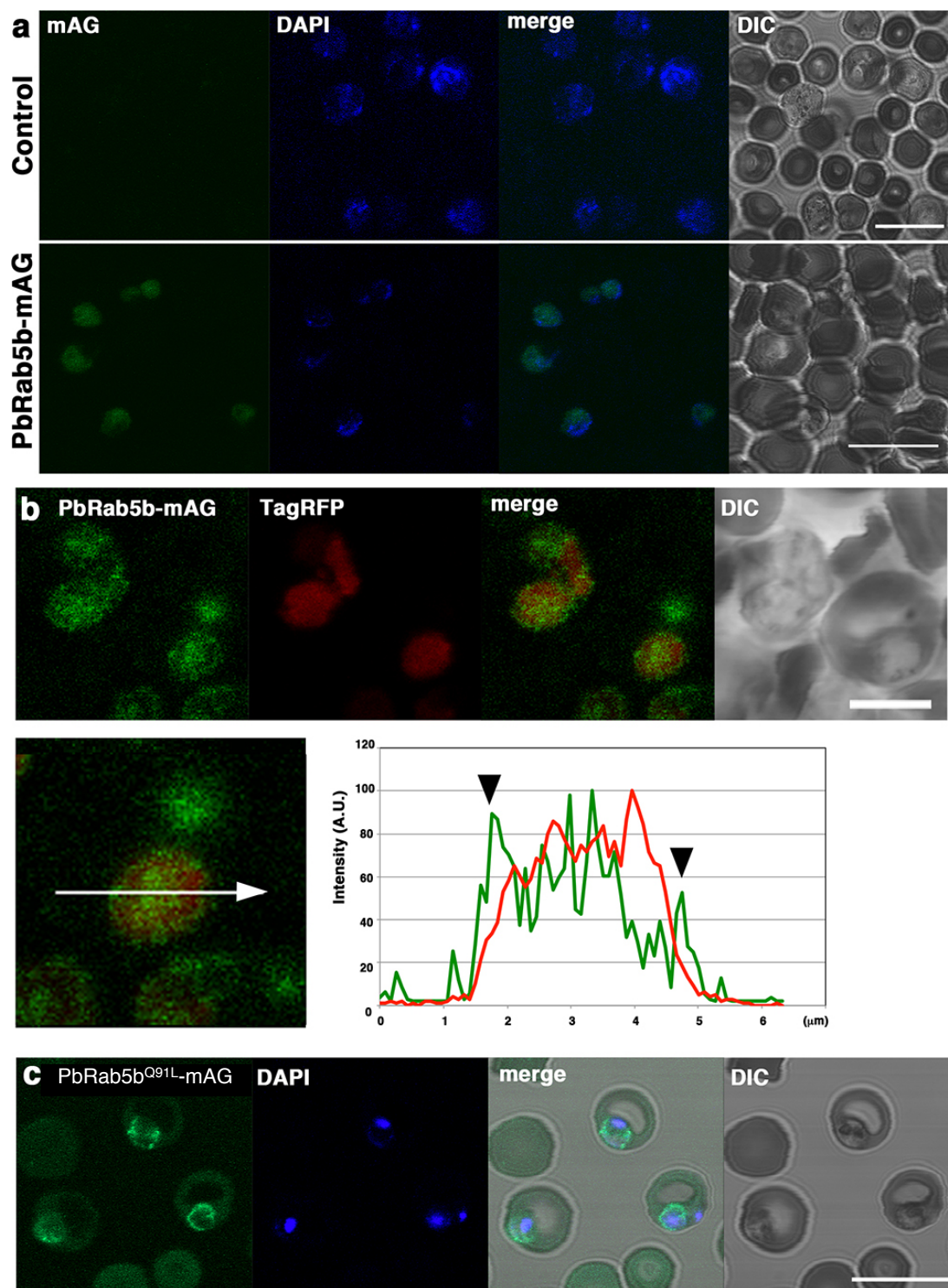

Supplement: Supplementary file 4 — 10.1186/s12936-016-1377-4 Peripheral localization of PbRab5b-mAG in trophozoite stage of parasites. (a) Fluorescence image of PbRab5b-mAG in trophozoite-stage parasites. Transgenic parasites expressing PbRab5b-mAG under the regulation of PbRab5b promoter were fixed with PFA, and faint cytosolic fluorescence of mAG were detected (green). Nuclei were stained with DAPI (blue). In control wild-type parasites, mAG fluorescence signal was not detected. Bar, 10 μm. (b) Magnified images of trophozoites expressing both PbRab5b-mAG and TagRFP. Trophozoite stage of parasites expressing PbRab5b-mAG (green) and cytosolic TagRFP (red) were fixed and the mAG and TagRFP fluorescence signals were obtained. Histograms of the green and red intensities along the white arrow are shown in the right graph. Black arrowheads indicate regions where stronger PbRab5b-mAG signal were detected compared to the TagRFP signal. Bar, 5 μm. (c) Peripheral localization of PbRab5bQ91L-mAG expressed under the regulation of PbRab5b promoter in trophozoite-stage parasites. A constitutively active PbRab5bQ91L-mAG mutant, in which Gln at aa 91 was replaced with Leu [1,2] was integrated into the upstream of PbRab5b genomic locus by single crossover method. Fluorescence of PbRab5bQ91L-mAG mutant protein was accumulated at the periphery of the parasite (green). Nuclei were stained with DAPI (blue). Bar, 5 μm.References[1] Li G, Barbieri MA, Colombo MI, Stahl PD (1994) Structural features of the GTP-binding defective Rab5 mutants required for their inhibitory activity on endocytosis. J Biol Chem 269: 14631-14635.[2] Stenmark H, Parton RG, Steele-Mortimer O, Lutcke A, Gruenberg J, et al. (1994) Inhibition of rab5 GTPase activity stimulates membrane fusion in endocytosis. EMBO J 13: 1287-1296. [file 12936_2016_1377_MOESM4_ESM.pdf]

**Figure S4**

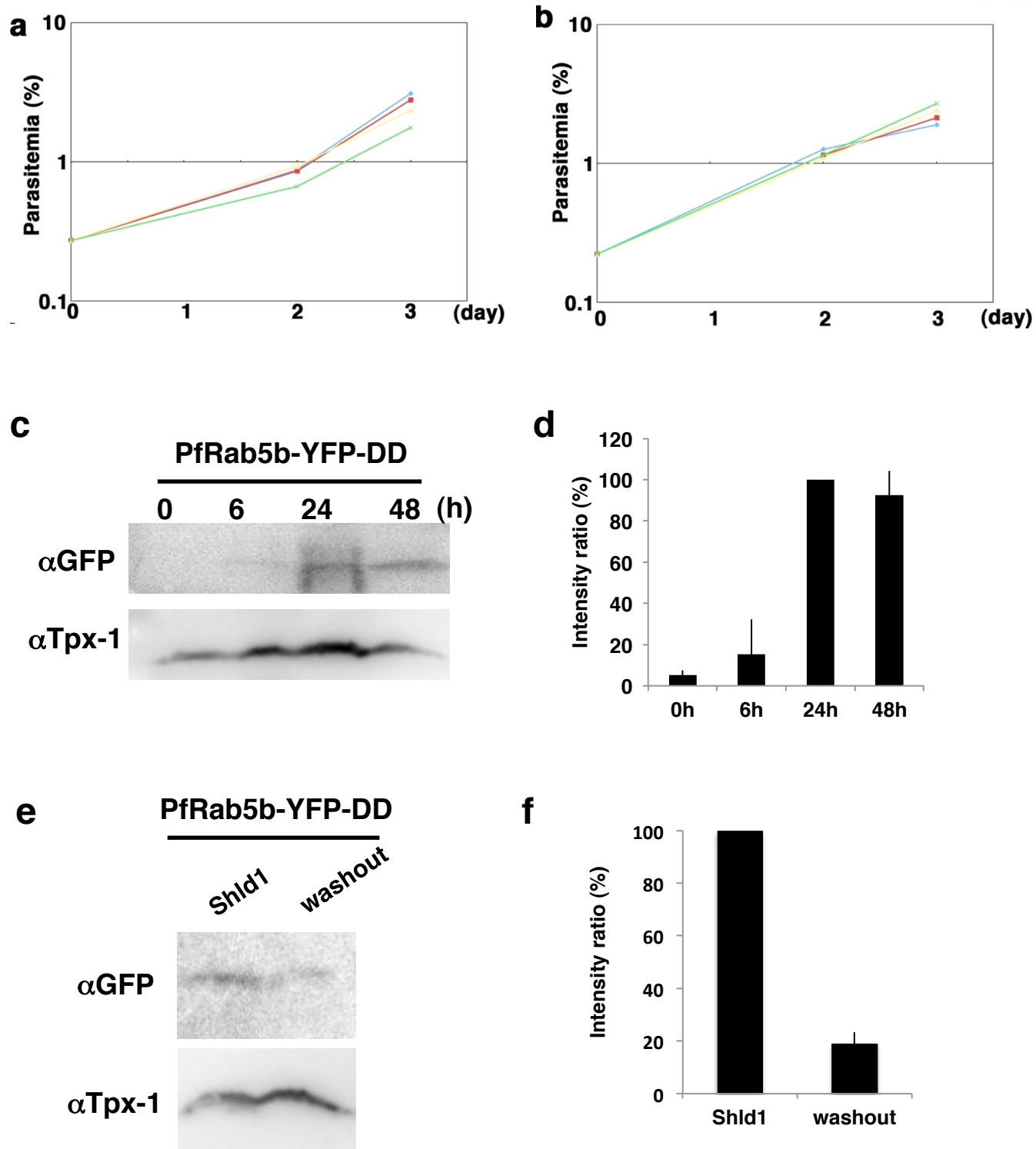

Supplement: Supplementary file 5 — 10.1186/s12936-016-1377-4 No growth defect by the expression of PfRab5b-YFP-DD or PfRab5bQ94L-YFP-DD seen in the growth curve. Growth curve of parasites expressing PfRab5b-YFP-DD (a) or PfRab5bQ94L-YFP-DD (b). Parasitemia (%) was measured after treatment with (green and yellow) or without (blue and red) 0.5 μM Shld1 in the presence of 5 nM WR99210-HCl. (c) Immunoblot using anti-GFP antibody to probe PfRab5-YFP-DD showed that PfRab5-YFP-DD was highly expressed after the incubation with 0.5 μM of Shld1 for 24 h. Tpx1 is a loading control. (d) Quantification of intensity of PfRab5-YFP-DD in Additional Figure 5c. (e) Immunoblot of PfRab5-YFP-DD before or after washout of Shld1 for 2 h. (f) Quantification of intensity of PfRab5-YFP-DD in Additional Figure 5e. [file 12936_2016_1377_MOESM5_ESM.pdf]

Figure S5

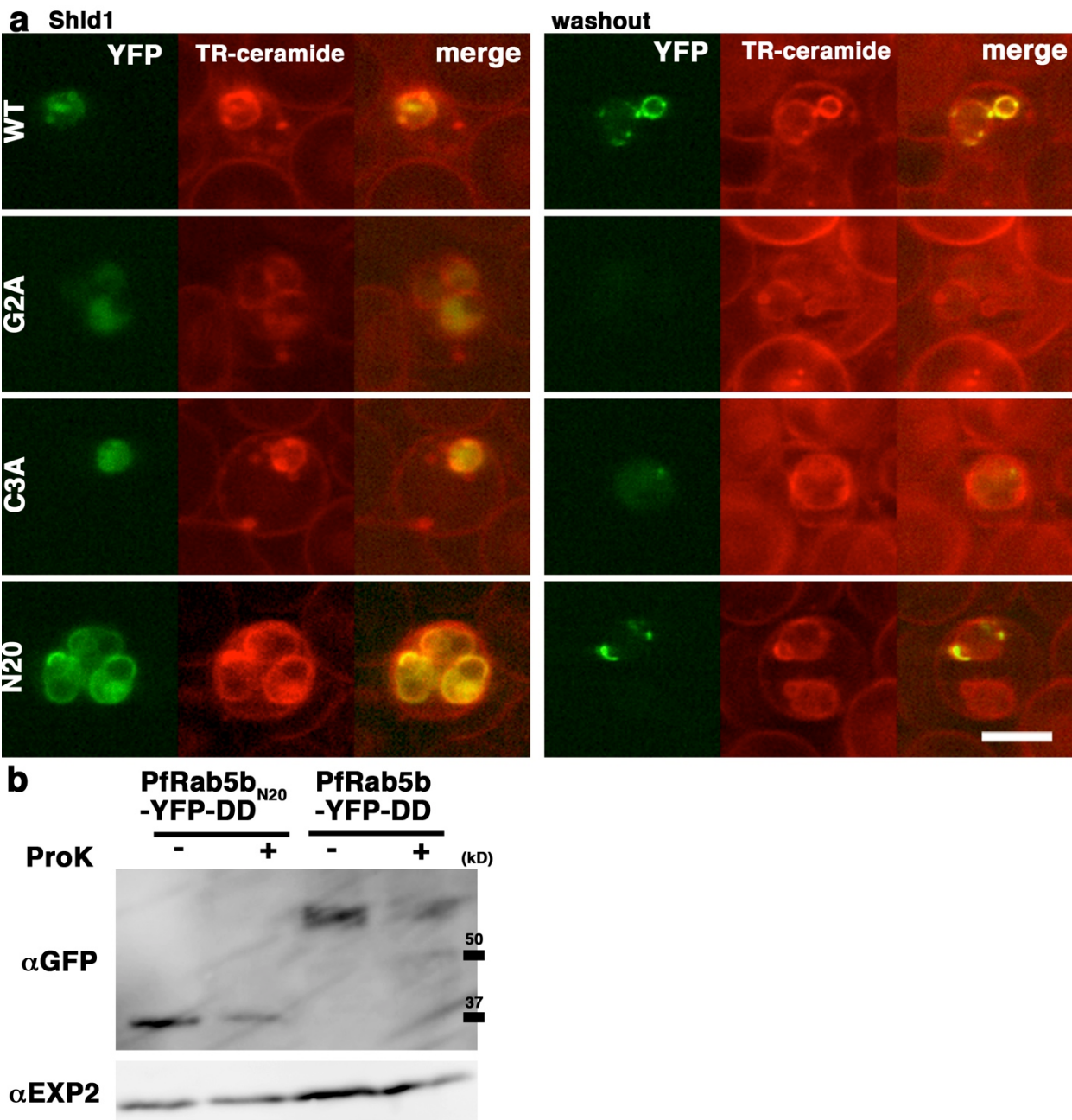

Supplement: Supplementary file 6 — 10.1186/s12936-016-1377-4 N-terminal myristoyl and palmitoyl modification of PfRab5b is essential for its transport to the TVN. (a) Subcellular localization of wild-type and mutated PfRab5b-YFP-DD (green) after 24-h Shld1 stabilization (left, Shld1) and after following 2-h Shld1 washout (right, washout). The TR-ceramide red fluorescent dye (red) labels the parasites and iRBC membranes. Washing out of Shld1 resulted in the loss of GFP signals from G2A and C3A mutants. (b) Immunoblotting after proteinase K (ProK) treatment of iRBCs permeabilized with streptolysin O. Anti-PfEXP2 antibody is used as a control which is not processed with proteinase K. [file 12936_2016_1377_MOESM6_ESM.pdf]
